# LAZARUS 1 functions as a positive regulator of plant immunity and systemic acquired resistance

**DOI:** 10.3389/fpls.2024.1490466

**Published:** 2024-11-20

**Authors:** Yue Chen, Yue Han, Weijie Huang, Yanjun Zhang, Xiaoli Chen, Dongyue Li, Yi Hong, Huhu Gao, Kewei Zhang, Yuelin Zhang, Tongjun Sun

**Affiliations:** ^1^ Shenzhen Branch, Guangdong Laboratory of Lingnan Modern Agriculture, Agricultural Genomics Institute at Shenzhen, Chinese Academy of Agricultural Sciences, Shenzhen, China; ^2^ Genome Analysis Laboratory of the Ministry of Agriculture and Rural Affairs, Agricultural Genomics Institute at Shenzhen, Chinese Academy of Agricultural Sciences, Shenzhen, China; ^3^ Department of Botany, University of British Columbia, Vancouver, BC, Canada; ^4^ Zhejiang Provincial Key Laboratory of Biotechnology on Specialty Economic Plants, College of Life Sciences, Zhejiang Normal University, Jinhua, Zhejiang, China; ^5^ Key Laboratory of Bio-resource and Eco-environment of Ministry of Education, College of Life Sciences, Sichuan University, Chengdu, China

**Keywords:** salicylic acid, N-hydroxypipecolic acid, CBP60g, SARD1, LAZ1

## Abstract

Systemic acquired resistance (SAR) is activated by local infection and confers enhanced resistance against subsequent pathogen invasion. Salicylic acid (SA) and N-hydroxypipecolic acid (NHP) are two key signaling molecules in SAR and their levels accumulate during SAR activation. Two members of plant-specific Calmodulin-Binding Protein 60 (CBP60) transcription factor family, CBP60g and SARD1, regulate the expression of biosynthetic genes of SA and NHP. CBP60g and SARD1 function as master regulators of plant immunity and their expression levels are tightly controlled. Although there are numerous reports on regulation of their expression, the specific mechanisms by which SARD1 and CBP60g respond to pathogen infection are not yet fully understood. This study identifies and characterizes the role of the LAZARUS 1 (LAZ1) and its homolog LAZ1H1 in plant immunity. A forward genetic screen was conducted in the *sard1-1* mutant background to identify mutants with enhanced SAR-deficient phenotypes (*sard* mutants), leading to the discovery of *sard6-1*, which maps to the *LAZ1* gene. LAZ1 and its homolog LAZ1H1 were found to be positive regulators of SAR through regulating the expression of *CBP60g* and *SARD1* as well as biosynthetic genes of SA and NHP. Furthermore, Overexpression of *LAZ1*, *LAZ1H1* and its homologs from *Nicotiana benthamiana* and potato enhanced resistance in *N. benthamiana* against *Phytophthora* pathogens. These findings indicate that LAZ1 and LAZ1H1 are evolutionarily conserved proteins that play critical roles in plant immunity.

## Introduction

Plant immunity relies on two major classes of immune receptors, located on the cell surface or intracellularly, which recognize a wide range of pathogens, including viruses, bacteria, fungi, oomycetes, insects, and nematodes, and activation of the plant’s immune system for self-defense (Jones et al., 2024; Man et al., 2022). The cell surface immune receptors, referred to as Pattern-Recognition Receptors (PRRs) detect pathogen-associated molecular patterns (PAMPs), activating pattern-triggered immunity (PTI), which restricts pathogen invasion (Jones and Dangl, 2006; Zhou and Zhang, 2020). Pathogens secrete effectors into host cells to suppress PTI and disrupt normal physiological processes, facilitating invasion (Jones and Dangl, 2006; Zhou and Zhang, 2020). Intracellular immune receptors, mainly a group of proteins with nucleotide-binding sites and leucine-rich repeat domains (NLRs), recognize effectors secreted by pathogens, activating effector-triggered immunity (ETI) (Jones and Dangl, 2006; Man et al., 2022; Zhou and Zhang, 2020). As a result, both PTI and ETI responses lead to the accumulation of defense signaling molecules, such as salicylic acid (SA) and N-hydroxypipecolic acid (NHP), and trigger secondary immune responses in distant tissues, known as systemic acquired resistance (SAR), which confer enhanced resistance against subsequent pathogen invasion (Chen et al., 2018; Fu and Dong, 2013; Hartmann and Zeier, 2019; Sun and Zhang, 2021).

SA and NHP are two plant defense signaling molecules involved in PTI, ETI and SAR (Hartmann and Zeier, 2019; Peng et al., 2021). Upon pathogen invasion, SA and NHP levels escalate in both local and systemic plant tissues (Hartmann and Zeier, 2019). Application of exogenous SA or NHP on plants enhances their disease resistance (Chen et al., 2018; Hartmann et al., 2018; Peng et al., 2021). In *Arabidopsis*, the perception of SA predominantly depends on the Non-expressor of *PR* genes 1 (NPR1) and its homologs, NPR1-LIKE proteins 3 and 4 (NPR3/4), leading to upregulating the expression of genes associated with immune responses (Ding et al., 2018; Fu et al., 2012; Wu et al., 2012). Although perception of SA by NPR1 and NPR3/NPR4 is required for NHP-induced resistance in *Arabidopsis*, NPR proteins fail to bind to NHP (Liu et al., 2020), implying that SA and NHP signaling might occur via distinct pathways.

Biosynthesis processes of both SA and NHP are well illustrated. SA biosynthesis in plants is mediated by the isochorismate synthase (ICS) and phenylalanine (Phe) ammonia-lyase (PAL) pathways (Peng et al., 2021). In *Arabidopsis thaliana*, the ICS pathway contributes predominantly to SA levels. The ICS pathway include ICS1, the MATE transporter EDS5 and the aminotransferase PBS3 (Rekhter et al., 2019; Torrens-Spence et al., 2019). ICS1 is the rate limiting enzyme of the ICS pathway and its expression level is tightly regulated by various transcription factors (Huang et al., 2020; Wildermuth et al., 2001). The NHP biosynthetic process involves three enzymatic steps performed by the aminotransferase ALD1, the reductase SARD4 and the monooxygenase FMO1, catalyzing the conversion of lysine into NHP (Chen S. et al., 2021; Ding et al., 2016; Navarova et al., 2012). Expression of these NHP biosynthetic genes is also dynamically controlled during plant defense (Huang et al., 2020).

Two members of plant-specific Calmodulin-Binding Protein 60 (CBP60) transcription factor family, CBP60g and SARD1, regulate expression of biosynthesis genes of both SA and NHP upon pathogen infection (Sun et al., 2015; Wang et al., 2009; Zhang et al., 2010). Despite their common ancestry within the same protein family, CBP60g and SARD1 operate through separate pathways. The loss of either SARD1 or CBP60g results in a significant reduction in the levels of *ICS1* and SA, while in the *sard1-1 cbp60g-1* double mutant, the induction of *ICS1* expression and the biosynthesis of SA are both blocked, suggesting that SARD1 and CBP60g regulate *ICS1* expression through two parallel pathways (Wang et al., 2011; Zhang et al., 2010). Expression of *SARD1* and *CBP60g* is also tightly regulated by various transcription factors, including positive regulators such as TGA1/4, NPR1, CBP60b, WRKY54/79 and GBPL3, as well as negative ones, including CAMTA1/2/3, NPR3/4 and HDA6 (Chen et al., 2021; Ding et al., 2018; Huang et al., 2021; Kim et al., 2022; Li et al., 2021; Sun et al., 2018, 2020; Wu et al., 2021). CBP60g is also regulated post-translationally. CALMODULIN (CAM) TOUCH3 and its homologs CAM1/4/6 cooperate with calcium-dependent protein kinases (CPK4/5/6/11) to phosphorylate and activate CBP60g (Sun et al., 2022). Although significant advances as mentioned above have been made, the specific mechanisms by which SARD1 and CBP60g respond to pathogen infection are not yet fully understood.

In pursuit of a deeper comprehension of CBP60g’s role in modulating plant immune responses, we conducted a forward genetic screen in *sard1-1* mutant background to look for mutants with enhanced *SAR-deficient* (*sard*) phenotype using the SAR assay developed by our group (Zhang et al., 2010). After two rounds of SAR screen, about 80 mutants show inheritable enhanced *sard* phenotype. The candidate mutants are further narrowed down to about 40 through direct sequencing known SAR genes, including *CBP60g*, *ICS1*, *EDS5*, *PBS3*, *ALD1* and *FMO1 etc*. In this study, we characterized and identified one of *sard1-1* enhancer mutants, namely *sard1-1 sard6-1*, using bulked-segregant analysis sequencing (BSA-Seq) and genetic complementation, confirming that *SARD6* encodes LAZARUS1 (LAZ1, AT4G38360). LAZ1 encodes a protein with a domain of unknown function (DUF300) and has been previously shown to modulate brassinosteroid and programmed cell death signaling pathways (Liu et al., 2018; Malinovsky et al., 2010). Here, we show that LAZ1 and its homolog LAZ1 HOMOLOG1 (LAZ1H1, AT1G77220) are positive regulators of plant immunity and SAR. In addition, LAZ1 and LAZ1H1 are conserved proteins and overexpression of their homologs from *Nicotiana benthamiana* (*Nb*) and *Solanum tuberosum* in *Nb* leaf showed enhanced resistance against *Phytophthora* pthogens. These results suggest that LAZ1 and LAZ1H1 are evolutionarily conserved and play a positive role in immunity.

## Materials and methods

### Plant material and growth environment

Arabidopsis plants were grown in soil at 23°C/21 °C day/night under 16/8-h light/dark cycles in a growth chamber with 40% relative humidity (RH) (Bi et al., 2010). The *N. benthamiana* plants were sowed and grown in a controlled environment room (CER) at 22 °C and 45–65% humidity with a 16/8-h light/dark cycles (Lin et al., 2023). Four-week-old plants were used for assay. The potato plants were grown in an artificial climate chamber at 25 ± 2 °C and 58–67% relative humidity under a 16/8-h light/dark photoperiod (Yang et al., 2023).

### Mapping‐by‐sequencing

Mapping‐by‐sequencing involves combining next-generation sequencing with classical genetic mapping to identify candidate mutations associated with a phenotype was carried out as previously described (Sun et al., 2020). The mutant phenotype of the selected F_2_ lines were confirmed by examining the self‐fertilized F_3_ progeny. Leaves were collected from the F_3_ progeny of 30 F_2_ lines with confirmed mutant-like phenotype. Genomic DNA was extracted from the mixed tissue and sent for WGS. WGS reads were aligned with the TAIR10 reference genome. SNPs were identified and the ratios of SNPs were plotted and used for linkage analysis. Genes containing nonsynonymous mutations in the linkage region were selected as candidate genes for knockout analysis.

### Mutant generation

The *laz1* mutations were generated by targeting *AT4G38360* in *sard1-1* and Col using the egg cell- specific promoter-controlled CRISPR/Cas9 system (Wang et al., 2015). The *laz1h1* mutations were generated by targeting *AT1G77220* in *sard1-1* and Col, respectively, using the same CRISPR/Cas9 system. The *sard1-1 sard6-2* F_2_ mutant was obtained by crossing *sard1-1* with SALK_023954C (*laz1-7*). The *sard1-1*mutant was reported (Zhang et al., 2010). Refer to **Supplementary Table S1** for a comprehensive list of all primers utilized in this process.

### Quantitative PCR

Total RNA was extracted from various tissues using TRIzol reagent (Invitrogen). Complementary DNAs (cDNAs) were synthesized using a ReverTra Ace kit (Toyobo) and served as templates for quantitative reverse transcription polymerase chain reaction (qRT-PCR), which was conducted with a SYBR Premix ExTaq kit (Takara) on a Bio-Rad iQ2 system. The procedure was as follows: initial polymerase activation for 30 s at 95°C followed by 40 cycles of 95°C for 5 s and 60°C for 20 s (Chen Y. et al., 2021). Each sample underwent three biological replicates and three technical replicates. The expression levels of the target genes were normalized to those of the actin gene. Primers used for qPCR can be found in **Supplementary Table S1**.

### Pathogen infection assays

The obligate pathogen *Hyaloperonospora arabidopsidis* (*Hpa*) Noco2 spore suspension in water was weekly propagated on Col seedlings at 18 °C and 60-80% humidity with a 12/12-h light/dark cycles (Zhang et al., 2010; Bi et al., 2010). The *Phytophthora infestans* (*P. infestans*) strain 1306 were cultured on Rye A agar medium at 18°C in the dark. The *Phytophthora capsici* (*P. capsici*) strain BYA5 was cultured on Rye A agar medium at 24°C in the dark (Abrahamian et al., 2016; Wang et al., 2019). The bacterial pathogen *Pseudomonas syringae pv maculicola* (*Psm*) ES4326 was cultured on King’s B medium Agar plate supplemented with 50 μg/mL streptomycin at 28°C incubator (Zhang et al., 2010).

For infection with *Psm* ES4326 (diluted in 10 mM MgCl_2_ to OD_600_ as indicated below) or 10 mM MgCl_2_, leaves of 3-week-old plants were infiltrated with the bacteria at a dose of OD_600_ = 0.0025-0.005 for SAR and OD_600_ = 0.001 for gene expression. For SAR assay (Zhang et al., 2010), the *Hpa* Noco2 infection assay was carried out on 3-week-old soil-grown seedlings two days after infection with *Psm* ES4326, by spraying plants with *Hpa* Noco2 spore suspension at a concentration of 5 x 10^4^ spores/mL. Inoculated plants were covered with a clean dome and grown at 18 °C under 12/12-h light/dark cycles in a growth chamber and growth of *Hpa* Noco2 was quantified seven days later. For genes expression, infected leaves were collected at two days after inoculation, two or three infected leaves of different plants were collected as one sample, and three samples were used for each genotype (Bi et al., 2010; Lan et al., 2023).

For inoculation assay with *P. infestans* strain 1306 (Abrahamian et al., 2016) and *P. capsici* strain BYA5 (Wang et al., 2019) on *Nb* leaves, Agrobacterium tumefaciens cultures were resuspended in infiltration buffer (10 mM MgCl_2_, 10 mM MES [pH 5.6], and 150 μM acetosyringone) at a final concentration of OD_600_ = 0.6 and infiltrated into leaves for transient expression of interested genes in planta. The leaves were detached 48h after agroinfiltration, then inoculated with *P. capsici* BYA5 mycelium (r = 2.5 mm) or 10 μL of the *P. infestans* 1306 zoospore suspension (200 zoospores/μL). The lesion areas (cm^2^) of *P. capsici*-inoculated leaves were measured under UV light at 48 h after inoculation. The *P. infestans*-inoculated leaves were incubated in a growth chamber at 18°C, and lesion areas were scored 3-4 days after infection.

### Determination of SA concentrations

The total SA was extracted following a modified method previously described for extraction of phenolic compounds (Zhang et al., 2012). About 100 mg of leaf tissue from 3- or 4-week-old plants 2 days after inoculation with *Psm* ES4326 (OD_600_ = 0.001) was collected, in four biological replicates from independent plants for each genotype. The rosette leaves were ground in liquid nitrogen. Around 100mg powders were added into 1ml 80% MeOH in a 2ml eppendorf tube. Then the eppendorf tube was agitated for 2hr at 4°C, and then centrifuged at 13,000g at 4°C for 10 min. The supernatant was transferred into a new eppendorf tube, and the sediment was re-extracted with 500μl 100% MeOH. Both extracts were combined and blow-dryed by nitrogen gas, then was resolved by 500μl sodium acetate (0.1M, pH 5.5). The resuspension was added with 10 μl β-glucosidase (1Uμl-1) and hydrolyzed at 37°C for 2hr in the water bath. After the hydrolysate was heated in boiling water for 5 min and centrifuged at 13,000g at 4°C for 10 min, the supernatant was used for analyzing total SA by HPLC as mentioned previously (Zhang et al., 2012). SA was detected at 296-nm excitation and 410-nm emission by using fluorescence detector. According to the standard curve, the concentration of SA is calculated by the HPLC peak area.

## Results

### Identification of *sard1-1 sard6-1* mutants

**Figure 1:**
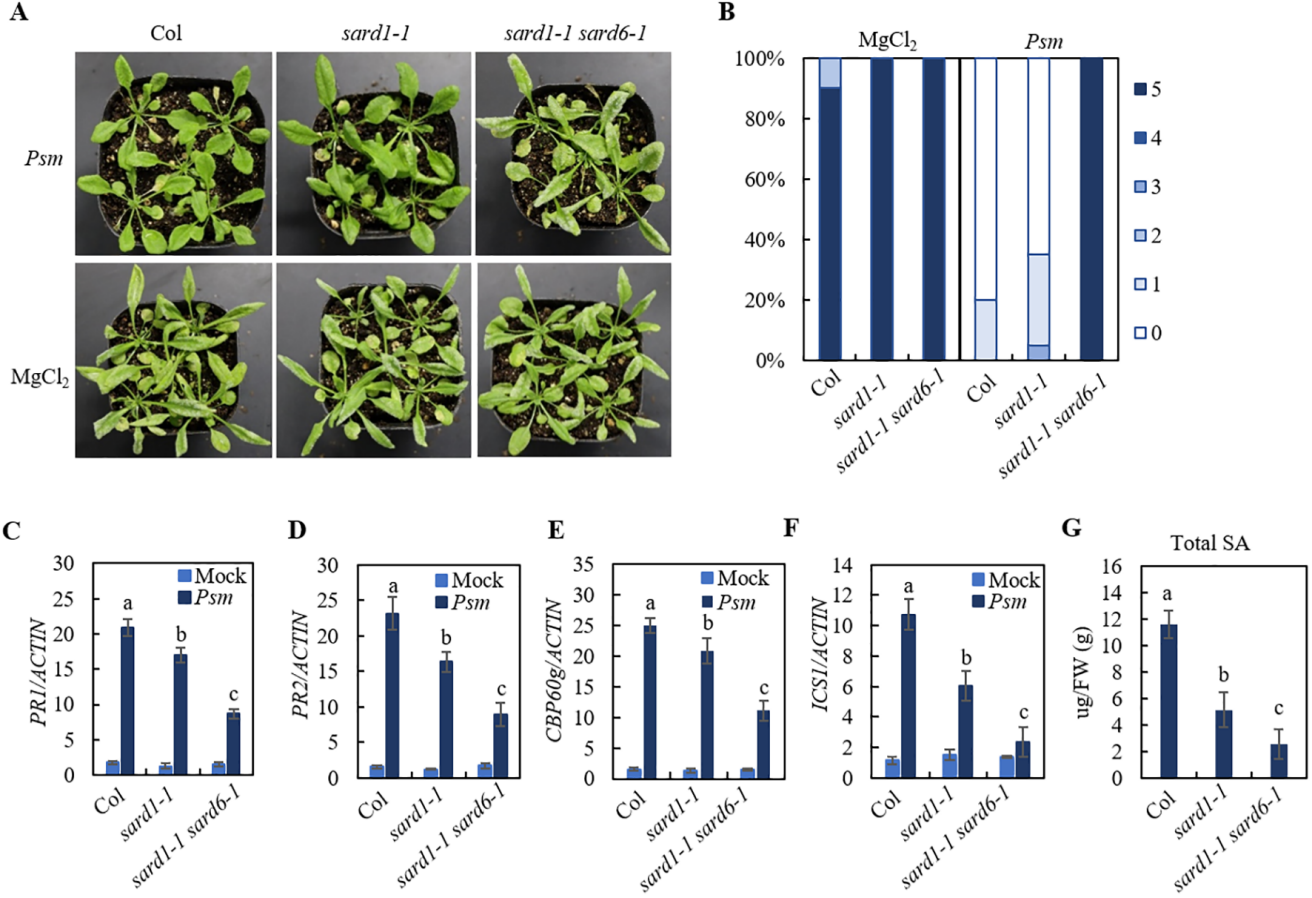
Identification of *sard1-1 sard6-1* mutant lines of Arabidopsis. **(A)** Growth of *Hpa* Noco2 on the distal leaves of wild-type Col, *sard1-1* and *sard1-1 sard6-1* plants in a SAR assay. Two primary leaves of 3-week-old plants were infiltrated with *Psm* ES4326 (OD_600_ = 0.0025) or 10 mM MgCl_2_ (mock) 2 d before the plants were sprayed with *Hpa* Noco2 spore suspension (50,000 spores/mL in water). **(B)** SAR phenotypic statistics of wild-type Col, *sard1-1* and *sard1-1 sard6-1* plants. Disease ratings are as follows: 0, no conidiophores on plants; 1, one leaf is infected with no more than five conidiophores; 2, one leaf is infected with more than five conidiophores; 3, two leaves are infected but with no more than five conidiophores on each infected leaf; 4, two leaves are infected with more than five conidiophores on each infected leaf; 5, more than two leaves are infected with more than five conidiophores. The experiment was repeated three times with independently grown plants, yielding similar results. **(C–F)** Expression of *PR1*, *PR2 CBP60g* and *ICS1*. Total RNA was extracted from the leaves of 3-week-old plants 2 d after infiltration with *Psm* ES4326 (OD_600_ = 0.001) or 10 mM MgCl_2_ (mock). Data were normalized relative to the expression of the *AtActin* gene. Error bars means ± SD of 3 biological replicates. Significant differences indicated by different letters were calculated using the Duncan’s new multiple range test. **(G)** total SA levels in leaves of Col, *sard1-1* and *sard1-1 sard6-1* 2 days after inoculation with *Psm* ES4326 (OD_600_ = 0.001). Bars represent means ± SD (n = 3). Statistically significant differences among the samples are labelled with different letters (one-way ANOVA with Tukey’s multiple comparisons test, P< 0.05).

As shown in **Figures 1A, B**, wild-type (Col) plants were susceptible to the virulent isolate of *Hyaloperonospora arabidopsidis* (*Hpa*) Noco2. After treatment with the bacterial pathogen *Pseudomonas syringae pv maculicola* (*Psm*) ES4326, Col plants became resistant against *Hpa* Noco2, suggesting an robust SAR response induced by *Psm* infection. The *sard1-1* plants showed a mild SAR-compromised phenotype, while the *sard1-1 sard6-1* double mutant exhibited an exacerbated SAR-deficient phenotype. We examined *Psm*-induced expression of the defense marker genes *PR1* and *PR2* in those lines and found that induction of both genes in *sard1-1 sard6-1* double mutant by *Psm* treatment was significantly reduced compared to that in *sard1-1* mutant (**Figures 1C, D**). We also detected a further compromised induction of critical genes involved in SA biosynthesis *CBP60g* and *ICS1* in the *sard1-1 sard6-1* mutant compared to *sard1-1* mutant (**Figures 1E, F**). Next, we quantified SA levels in Col, *sard1-1* and the *sard1-1 sard6-1* mutant plants. Following treatment with *Psm*, the total SA levels in the *sard1-1 sard6-1* mutant was significantly reduced compared to those in the Col or *sard1-1* plants (**Figure 1G**). These findings indicate that the systemic resistance in the *sard1-1 sard6-1* mutant may be impeded due to the impact on the induction of *CBP60g* and SA biosynthesis.

### *SARD6* encodes LAZARUS1

**Figure 2:**
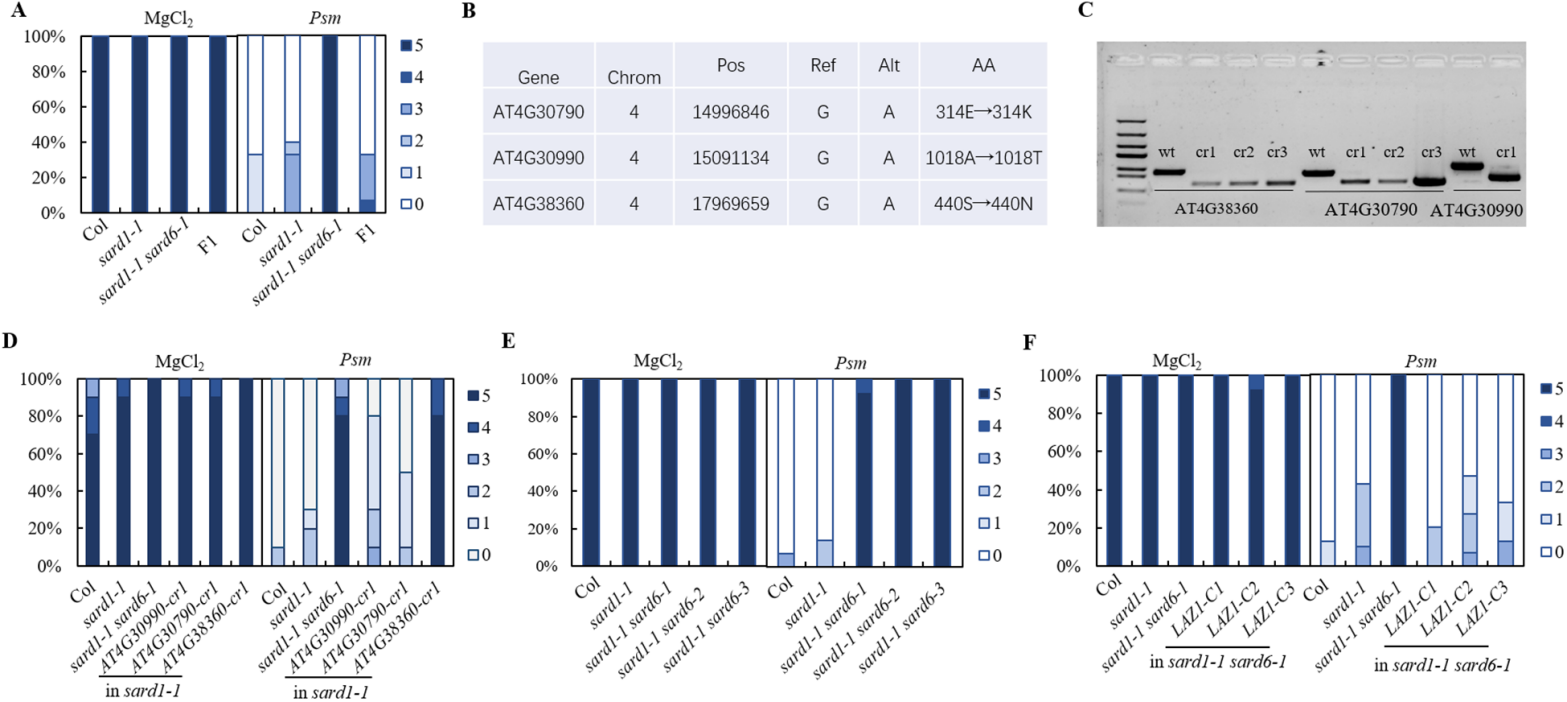
Positional cloning and gene verification of *sard6.* **(A)** SAR phenotypic statistics of wild-type Col, *sard1-1*, *sard1-1 sard6-1* double mutants and the F_1_ progeny of *sard1-1* crossed with *sard1-1 sard6-1* plants. **(B)** Compilation of candidate genes with annotations including Chromosome (Chrom), Position (Pos), Reference allele (Ref), Alternate allele (Alt), and Amino acid (AA) changes. **(C)** Electropherograms depicting the wild-type and knockout genotypes for the candidate genes. The wild-type (WT) lanes are aligned alongside those representing the respective deletion mutants generated using CRISPR/Cas9 system. cr1 lines are in *sard1-1* background and used in **Figure 1D**. **(D)** SAR phenotypic statistics comparing the wild-type Col, *sard1-1*, *sard1-1 sard6-1* double mutants, and deletion lines in *sard1-1* background: *AT4G30990*-*cr1* (deletion mutation in *AT4G30990*), *AT4G30790*-*cr1* (deletion in *AT4G30790*), and *AT4G38360*-*cr1* (deletion in *AT4G38360*). **(E)** SAR phenotypic statistics of wild-type Col, *sard1-1*, *sard1-1 sard6-1* and *sard1-1 sard6-2*, *sard1-1 sard6-3* plants. *sard6-2* and *sard6-3* are T-DNA allele and deletion allele for *AT4G38360*, respectively; see details in **Supplementary Figures S2A**, **B**. **(F)** SAR phenotypic statistics of wild-type Col, *sard1-1*, *sard1-1 sard6-1* and *sard1-1 laz1-C* plants. *sard1-1 laz1-C*: complementation lines of AT4G38360 in *sard1-1 sard6-1*.

To identify *sard6*, we performed combining next-generation sequencing with classical genetic mapping to identify candidate mutations associated with a phenotype on *sard1-1 sard6-1* mutant. The *sard1-1 sard6-1* mutant was backcrossed with the *sard1-1* line and resulting F_1_ plants exhibit *sard1-*like *sard* phenotype (**Figure 2A**; **Supplementary Figure S1A**), suggesting *sard6-1* is a recessive mutant. In the F_2_ generation, lines with *sard1-1 sard6-1-*like and *sard1-*like *sard* phenotype were kept and validated in the F3 progeny, respectively. Pooled genomic DNA from each segregant population (30 confirmed lines) was subjected to whole-genome next-generation sequencing (WGS). Analysis of the single nucleotide polymorphism (SNP) frequency distribution across the genome unveiled a linked genetic region on chromosome 4 (**Supplementary Figure S1B**). Within this chromosomal segment, three genes, *AT4G30790*, *AT4G30990* and *AT4G38360*, using linkage analysis, we detected G to A transitions that resulted in missense mutations (**Figure 2B**). To ascertain the gene correlating with the *sard1-1 sard6-1* phenotype, we generated deletion mutant for each gene in *sard1-1* background using CRISPR/Cas9 technology (**Figure 2C**). Upon subsequent SAR analysis of the homozygous lines for the three candidate genes, only *sard1-1 AT4G38360*-cr double mutant phenocopied *sard1-1 sard6-1*, indicating that *AT4G38360*, alias *LAZARUS1* (*LAZ1*) (Malinovsky et al., 2010), is the gene of interest (**Figure 2D**).

To further ascertain the association of the mutation in *AT4G38360* with the *sard1-1 sard6-1* phenotype, we identified the *sard1-1 sard6-2* double mutant from F_2_ progeny of a cross between *sard1-1* and *sard6*-2, a T-DNA mutant SALK_023954C targeting the *At4G38360* locus and silencing the gene (**Supplementary Figures S2A, S2C**), and confirmed DNA fragment deletion in *sard1-1 AT4G38360*-cr double mutant (reassigned as *sard1-1 sard6*-3) using Sanger sequencing (**Supplementary Figure S2B**). Subsequent SAR verification revealed that both *sard1-1 sard6-2* and the *sard1-1 sard6-3* lines exhibited *sard1-1 sard6-1*-like *sard* phenotype (**Figure 2E**). Additionally, we performed genetic complementation by agrobacteria mediated transformation of a 4.3 kb fragment containing LAZ1 coding region into the *sard1-1 sard6-1* mutant. Three independent lines with LAZ1 expression were chosen and tested for SAR phenotype (**Supplementary Figure S2D**). As shown in **Figure 2F**, these three lines exhibited *sard1*-like *sard* phenotype, indicating that expressing LAZ1 revert the enhanced *sard* phenotype of *sard1-1 sard6-1*. These findings support that the *SARD6* locus corresponds to *AT4G38360*/*LAZ1*, encoding a protein with a DUF300 domain. This protein is implicated in vacuolar transport and appears to modulate brassinosteroid signaling pathways (Malinovsky et al., 2010). For simplicity and consistency, *sard6-1*, *sard6-2* and *sard6-3* is reassigned to *laz1-6*, *laz1-7 and laz1-8*, respectively (**Supplementary Figure S2A**).

### *LAZ1 HOMOLOGO1* positively regulates SAR

In Arabidopsis, LAZ1 has a close homolog, LAZ1 Homolog 1 (LAZ1H1, AT1G77220). Quantitative PCR analysis of the expression of *LAZ1* and *LAZ1H1* showed that both genes were induced after *Psm* ES4326 treatment (**Supplementary Figures S3A, B**), indicating that, like *LAZ1*, *LAZ1H1* may also play a role in plant immunity. To check whether LAZ1H1 contributes to SAR, we employed CRISPR/Cas9 technology to generate knockout mutants for *LAZ1H1* in both the Col and *sard1-1* backgrounds. Utilizing PCR amplification and sanger sequencing, we identified three homozygous deletion lines in the *sard1-1* background and three deletion lines in Col, designated as *sard1-1 laz1h1-1*, *sard1-1 laz1h1*-*2*, *sard1-1 laz1h1*-*3*, *laz1h1-4*, *laz1h1-5* and *laz1h1-6*, respectively (**Supplementary Figure S3C**). Upon verification of SAR response in the *sard1-1 laz1h1* lines, we observed that they exhibited the same phenotype as the *sard1-1 laz1-8* double knockout, while *laz1h1* mutants showed minimal *sard* phenotype (**Supplementary Figure S3D**), suggesting that LAZ1H1 also positively regulates SAR.

### LAZ1 and LAZ1H1 play overlapping roles in plant immunity

**Figure 3:**
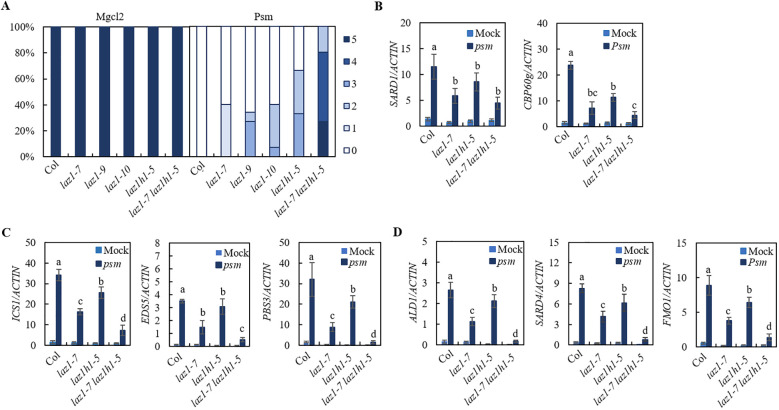
SAR phenotype in *laz1* and *laz1h1* mutants. **(A)** SAR phenotypic statistics in Col, *laz1-7*, *laz1-9*, *laz1-10*, *laz1h1-5* and *laz1-7 laz1h1-5* plants. **(B–D)** Expression of *SARD1*, *CBP60g*, *ICS1*, *EDS5*, *PBS3*, *ALD1*, *SARD4* and *FMO1*. Total RNA was extracted from the leaves of 3-week-old plants 2d after infiltration with *Psm* ES4326 (OD_600_ = 0.001) or 10 mM MgCl_2_ (mock). Data were normalized relative to the expression of the *AtActin* gene. Error bars means ± SD of 3 biological replicates. Significant differences indicated by different letters were calculated using the Duncan’s new multiple range test.

To investigate the roles of *LAZ1* and *LAZ1H1* in SAR, we employed CRISPR/Cas9 technology to generate knockout mutants for *laz1* in Col background. Utilizing PCR amplification and sanger sequencing, we identified two homozygous deletion lines in Col, designated as *laz1-9*, *laz1-10* respectively (**Supplementary Figure S2B**), and generated *laz1-7 laz1h1-5* double mutant from F_3_ progeny of a cross between *laz1-7* and *laz1h1-5*. *Psm*-induced SAR in Col, *laz1-7*, *laz1-9*, *laz1-10*, *laz1h1-5*, *laz1-7 laz1h1-5* plants were performed. Compared to Col, the mutants *laz1-7*, *laz1-9* and *laz1*-*10*, *laz1h1-5* exhibited a weak *sard* phenotype, *laz1-7 laz1h1-5* exhibited a stronger *sard* phenotype (**Figure 3A**).

We examined *Psm*-induced expression of *SARD1 and CBP60g* as well as biosynthetic genes of SA and NHP in Col, *laz1-7*, *laz1h1-5* and *laz1-7 laz1h1-5* lines and found that induction of these genes in three mutants were significantly reduced compared to that in Col, and the expression levels of *ICS1*, *EDS5*, *PBS3*, *ALD1*, *SARD4* and *FMO1* were lower in *laz1-7 laz1h1-5* (**Figures 3B–D**). The results showed that *laz1* and *laz1h1* play overlapping roles in plant immunity.

### *LAZ1* is not required for NHP-induced immunity

Given that NHP acts as the mobile signal for SAR (Chen et al., 2018; Hartmann et al., 2018) and that the LAZ1/LAZ1H1 are putative channel proteins, we investigated whether LAZ1/LAZ1H1 is necessary for NHP-induced immune responses. Col, along with the *laz1-7*, *laz1-9*, *laz1-10*, *laz1h1-4*, *laz1h1-5*, *laz1h1-6* and *laz1-7 laz1h1-5* mutants were utilized as experimental materials for verification. Initially, we infiltrated the primary leaves with 1 mM or 0.3 mM NHP and subsequently spray-inoculated the entire plants with a spore suspension of *Hpa* Noco2, separately. As depicted in **Supplementary Figure S4**, minimal pathogen growth was observed on Col pretreated with NHP, indicating that NHP confers robust resistance against *Hpa* Noco2. Similar outcomes were observed in the NHP-pretreated seven mutant lines. These results suggest that *laz1* and *laz1h1* are not involved in the regulation of NHP-induced immunity.

### Overexpression of *LAZ1* and *LAZ1H1* enhances the resistance of *N. benthamiana* to *Phytophthora*

**Figure 4:**
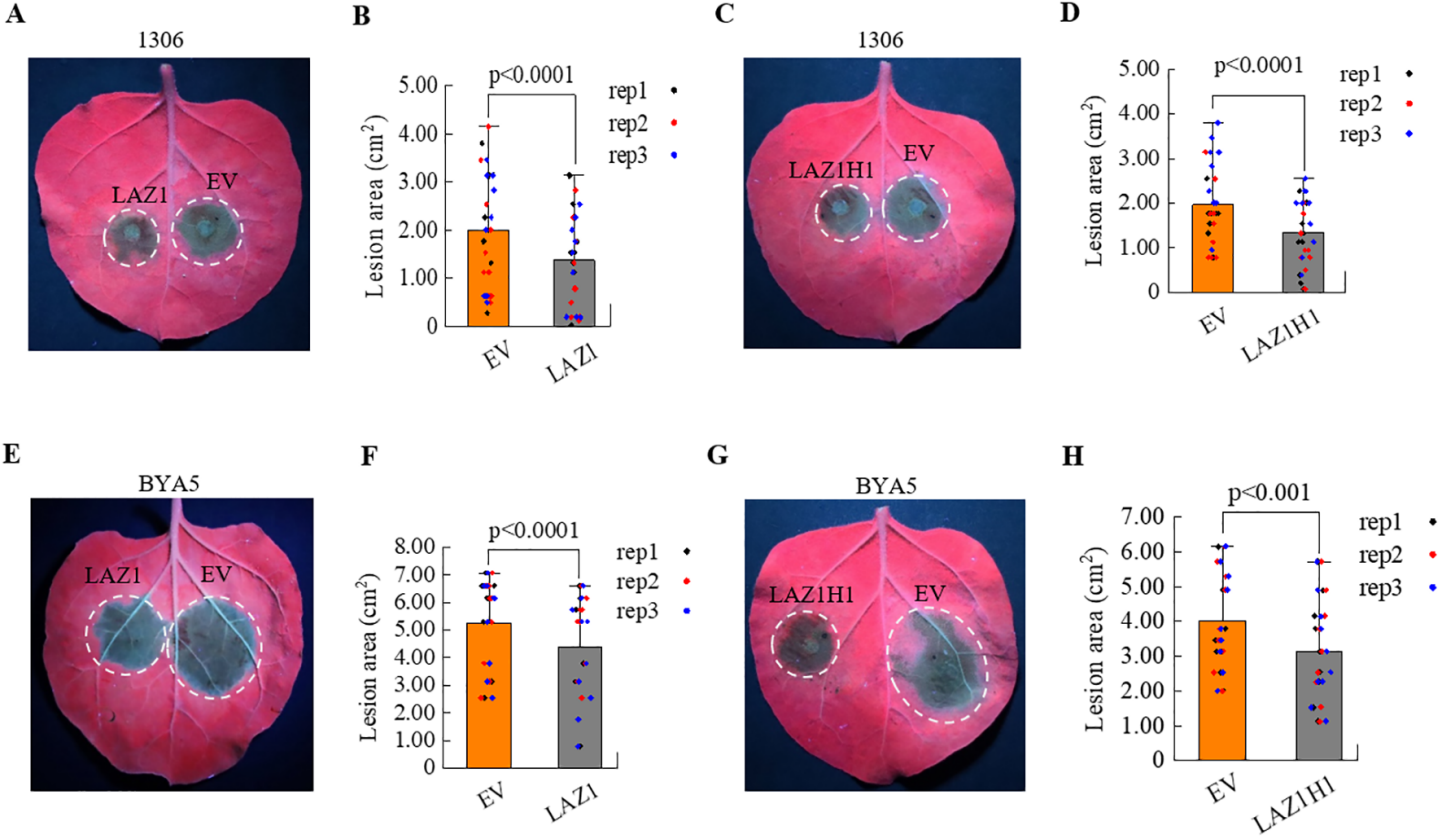
Impact of *LAZ1* and *LAZ1H1* on *N. benthamiana*. **(A)** Infection assays on *N. benthamiana* (*Nb*) leaves transient expression of LAZ1 or EV with *P. infestans* strain 1306. Two days after infiltration with Agrobacterium carrying *35S-LAZ1* or empty vector (EV), leaf areas were inoculated with *P. infestans* 1306 zoospore suspension. Detached leaves were incubated in a growth chamber at 18°C, and lesion areas were scored under UV light 3–4 days after infection. **(B)** Statistical analyses on lesion sizes of *Nb* leaves in **Figure 4A** (n=10-15 from three replicates). **(C)** Infection assays on *Nb* leaves transient expression of LAZ1H1 or EV with *P. infestans* strain 1306, following the same procedure as in **Figure 4A**. **(D)** Statistical analyses on lesion sizes of *Nb* leaves in **Figure 4C** (n=10-15 from three replicates). **(E–H)** Infection assays on *Nb* leaves transient expression of LAZ1, LAZ1H1 or EV with *P. capsici* strain BYA5. Two days after infiltration with Agrobacterium carrying *35S-LAZ1* **(E)**, *35S-LAZ1H1* **(G)** or empty vector (EV), leaf areas were inoculated with *P. capsici* BYA5 mycelium. Detached leaves were incubated in a growth chamber at 25°C, and lesion areas were measured under UV light 36 h after inoculation. Statistical analyses on lesion sizes were shown in **(F)** (n=10-15 from three replicates) and **(H)** (n=10-15 from three replicates), respectively. In **(B, D, F, H)** data were normally distributed and were shown as means ± SD. Outliers were identifed and removed using Grubbs test. Statistical significance was determined by Student’s *t* test.

The results mentioned above suggest that LAZ1 and LAZ1H1 positively regulate immunity against the obligate oomycete *Hpa* Noco2. To determine if LAZ1 and LAZ1H1 could augment resistance against different *Phytophthora* pathogens, we individually inserted the genomic sequences of *LAZ1* and *LAZ1H1*, including 35S promoters, coding regions, and terminators, into the pCAMBIA1300 vector. These constructs were transformed into agrobacterium and used for agrobacterium-mediated transient overexpression of the respective proteins in *Nicotiana benthamiana* (*Nb*) leaves. After 48 hours post-infiltration of agrobacterium strain carrying 35S-*LAZ1*, 35S-*LAZ1H1* or empty vector, the *Nb* leaves were inoculated with spores of *P.infestans* strain 1306 or mycelium of *P.capsici* strain BYA5. The resulting lesion areas were evaluated 3-4 days following infection. In **Figure 4A**, *Nb* leaf areas overexpressing *LAZ1* exhibited significantly reduced lesion sizes compared to those with an empty-vector (EV) control after inoculation with *P.infestans* strain 1306, with statistically significant differences in lesion sizes observed (**Figure 4B**). Overexpression of *LAZ1H1* yielded analogous results (**Figures 4C, D**).

Similarly, overexpression of *LAZ1* or *LAZ1H1* lead to reduced lesion sizes compared to the EV control, following infection with *P.capsici* strain BYA5 (**Figures 4E–H**). These findings indicate that overexpression of *LAZ1* or *LAZ1H1* in *Nb* leaves can enhance resistance to *P.infestans* and *P.capsici*.

### Overexpression of homologous genes of *LAZ1* enhances the resistance of *N. benthamiana* to *P.infestans*

**Figure 5:**
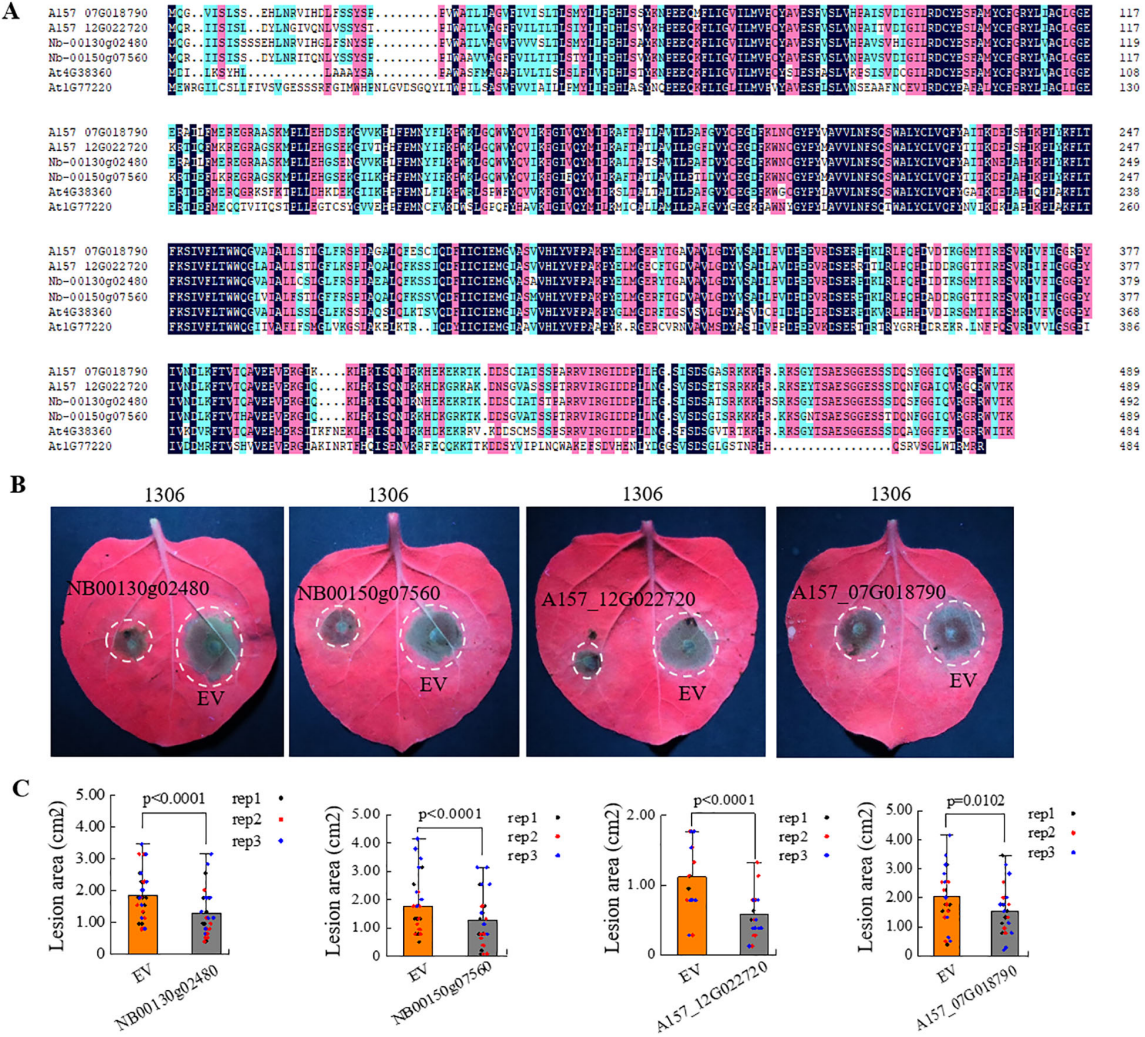
Influence of *LAZ1* homologous genes from *N. benthamiana* and potato on *N. benthamiana* immunity. **(A)** Protein sequence alignment with the highest homology of LAZ1 in *N. benthamiana* and potato. **(B)** Infection assays were performed on *Nb* leaves overexpressing the *LAZ1* homologous genes from *N. benthamiana* (*NB00130g02480* and *NB00150g07560*) and potato (*A157_12G022720* and *A157_07G018790*), challenged with *P. infestans* strain 1306. **(C)**, Data processing and statistical analysis of lesion sizes in **(B)**. Data were normally distributed and were shown as means ± SD (n=10-15 from three replicates). Outliers were identified and removed using Grubbs test. Statistical significance was determined by Student’s *t* test.

Homologs of *LAZ1* were found in various plants. To understand whether homologs of the *LAZ1* gene in *N.benthamiana* and *Solanum tuberosum* (potato) can enhance resistance to *Phytophthora*, we identified sequences with high homology to *LAZ1* from the *N.benthamiana* genome database (https://nbenthamiana.jp/nbrowser/anno) (Kurotani et al., 2023) and the genome database of the diploid potato inbreeding line A157 (Zhang et al., 2021). The protein sequences of Nbe.v1.s00130g02480 (NB00130g02480) and Nbe.v1.s00150g07560 (NB00150g07560) in *N.benthamiana* and the protein sequences of A157_07G018790 and A157_12G022720 in A157 showed the highest homology to LAZ1 protein (**Figure 5A**). Phylogenetic analysis grouped these four genes into a single cluster, suggesting a close evolutionary relationship, and LAZ1 is closely related to these four proteins (**Supplementary Figure S5A**). The coding sequences (CDS) of these four homologs gene were amplified and individually inserted into the binary pCAMBIA1300 vector under the 35S promoter. Transient expression in *Nb* leaves followed by inoculation with *P.infestans* strain 1306 revealed that the lesion areas at sites of overexpression of these four genes were significantly reduced compared to the empty-vector (EV) control (**Figure 5B**), with statistically significant differences observed (**Figure 5C**). Validation through western blot analysis confirmed the presence of protein products encoded by these genes in *Nb* leaves (**Supplementary Figure S5B**). These findings indicate that overexpression of these *LAZ1* homologs significantly enhances the resistance of *N. benthamiana* to *P.infestans*.

## Discussion

### LAZ1/LAZ1H1 are positive regulators of SAR

LAZ1 and LAZ1H1 belong to the evolutionarily conserved DUF300 family of transmembrane proteins in eukaryotes. LAZ1 serves as a regulatory factor for certain Hypersensitive Response (HR) cell deaths conditioned by the TIR-NB-LRR protein RPS4 and by the CC-NB-LRR protein RPM1 (Malinovsky et al., 2010). LAZ1 and LAZ1H1 have been shown to play a pivotal role in maintaining vacuole membrane integrity, which is crucial for proper Brassinosteroid (BR) signaling (Liu et al., 2018). Despite these insights, the roles of LAZ1 and LAZ1H1 in the systemic acquired resistance (SAR) pathway remain unexplored. In this report, we show that LAZ1 and LAZ1H1 are positive regulators of SAR.

Employing a forward genetic strategy, we isolated the *sard1-1 sard6-1* mutant as one of *sard* enhancers of *sard1-1*. This *sard1-1 sard6-1* mutant exhibited severely impaired systemic resistance (**Figures 1A, B**). Through mapping-by-sequence and gene complementation, we identified that *SARD6* encodes LAZ1 (**Figure 2**). We also found that loss-of-function of *LAZ1H1* leads to enhanced SAR deficiency in *sard1-1* background (**Supplementary Figure S3**). Furthermore, we showed that the *laz1 laz1h1* double mutant exhibited stronger *sard* phenotype compared to *laz1* and *laz1h1* single mutants (**Figure 3A**), indicating a functional redundancy between LAZ1 and LAZ1H1 in regulation of SAR.

In this study, we have used *Psm* ES4326, a virulent pathogen which does not trigger HR, to induce SAR and found that *Laz1* and *laz1h1* mutants are compromised in SAR, suggesting that LAZ1/LAZ1H1 play an HR-independent role in SAR.

### LAZ1/LAZ1H1 regulate SAR by affecting expression of biosynthesis genes of SA and NHP

SA and NHP are two key signaling molecules in SAR and expression levels of their biosynthetic genes are tightly controlled during plant immunity (Hartmann and Zeier, 2019; Peng et al., 2021). SARD1 and CBP60g are master transcriptional regulators in plant defense and positively regulate biosynthetic genes of SA and NHP (Sun et al., 2020). *Psm*-induced expression of *CBP60g* and *ICS1* in *sard1-1 sard6-1* was further diminished compared to that in *sard1-1* (**Figures 1E, F**) and total SA levels was lower in *sard1-1 sard6-1* than that in *sard1-1* (**Figure 1G**), indicating that the involvement of LAZ1 in SAR may be attributed to its regulatory effects on the expression of *CBP60g* and *ICS1*, as well as the accumulation of SA.

*laz1* and *laz1h1* single mutants showed minor *sard* phenotype while *laz1 laz1h1* double mutant exhibited stronger *sard* phenotype (**Figure 3A**). Accordingly, *Psm*-induced expression of biosynthesis genes of SA and NHP in *laz1* and *laz1h1* single mutants was reduced compared to that in Col, and further diminished in *laz1 laz1h1* double mutant (**Figures 3C, D**), suggesting that LAZ1 and LAZ1H1 have an overlapping function in SAR and that they positively regulate SAR by modulating the expression of biosynthetic genes of SA and NHP. Since *Psm*-induced expression of *SARD1* and *CBP60g* was diminished in *laz1 laz1h1* double mutant (**Figure 3B**), it is possible that LAZ1 and LAZ1H1 modulate the expression of biosynthetic genes of SA and NHP through regulating expression of *SARD1* and *CBP60g*. In addition, we showed that NHP-induced resistance against *Hpa* Noco2 was not significantly affected in *laz1*, *laz1h1 or laz1 laz1h1* double mutant (**Supplementary Figure S4**). These results suggest that LAZ1 and LAZ1H1 regulate SAR mainly through affecting the expression of biosynthesis genes of SA and NHP. However, the underlying mechanism necessitates further investigation.

Recently, LAZ1 homologs in maize, ZmLAZ1-4 and ZmLAZ1-8, were predicted to bind metal ions including Zn^2+^, Mg^2+^, or Ca^2+^ and ZmLAZ1-4 protein was shown to act as a Zinc transporter that modulate Zinc homeostasis on plasma and vacuolar membrane (Liu et al., 2022), it will be interesting to test whether LAZ1 and LAZ1H1 combine Ca^2+^ and regulate the expression of defense genes through modulating calcium homeostasis during plant immunity.

### LAZ1 and LAZ1H1 are evolutionarily conserved

LAZ1 and LAZ1H1 are conserved proteins with their homologs found in various plants. We showed overexpression of *LAZ1* and *LAZ1H1* as well as their close homologs from *N.benthamiana* and potato in *Nb* leaves leads to enhanced resistance to *Phytophthora* species (**Figures 4**, **5**), suggesting that LAZ1 and LAZ1H1 are evolutionarily conserved in positive regulation of plant defense, thereby emphasizing the significance and utility of these genes in investigating plant-pathogen interactions. Presently, in potato, the cultivation of disease-resistant varieties is mainly to isolate disease resistance genes from wild species and introduce them into cultivated varieties through genetic transformation. The homologs of LAZ1 in potato were found to similarly bolster the plants’ resistance to Phytophthora (**Figures 5B, C**). This study provides an important genetic resource for potato disease resistance breeding.

## Data Availability

The original contributions presented in the study are included in the article/**Supplementary Material**. Further inquiries can be directed to the corresponding author.
