# Supplementary material for: LAZARUS 1 functions as a positive regulator of plant immunity and systemic acquired resistance
Source: Front Plant Sci. 2024 Nov 20;15:1490466. doi: 10.3389/fpls.2024.1490466 (PMC11614604; doi:10.3389/fpls.2024.1490466)
Supplement: Supplementary file 1 [file DataSheet1.pdf]

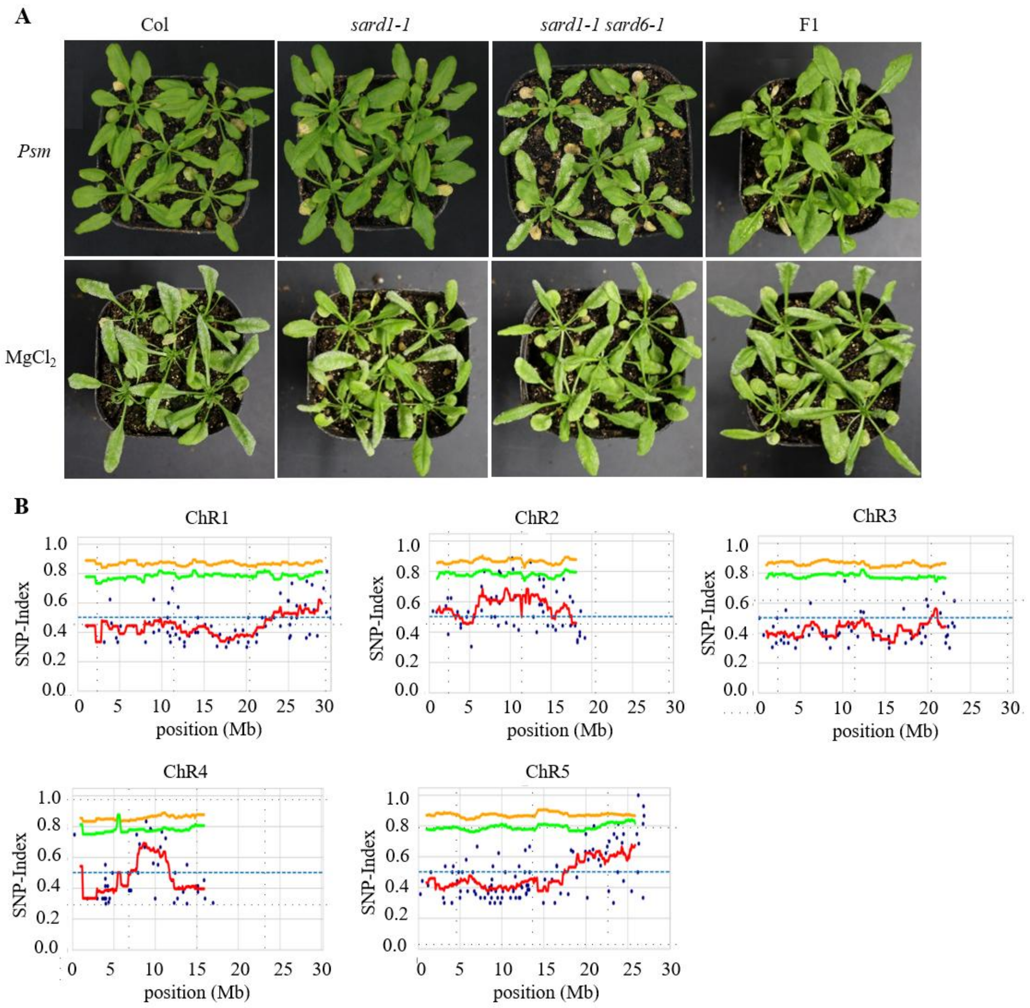

Figure S1. **SAR validation of mutants and whole genome next generation sequencing analysis.**

A, Growth of *Hpa* Noco2 on the whole plant of wild-type Col, *sard1-1*, *sard1-1 sard6-1* and F1 plants in the SAR assay. **B**, Analysis of the single nucleotide polymorphism (SNP) frequency distribution throughout the genome identified a genetic linkage region on chromosome4.

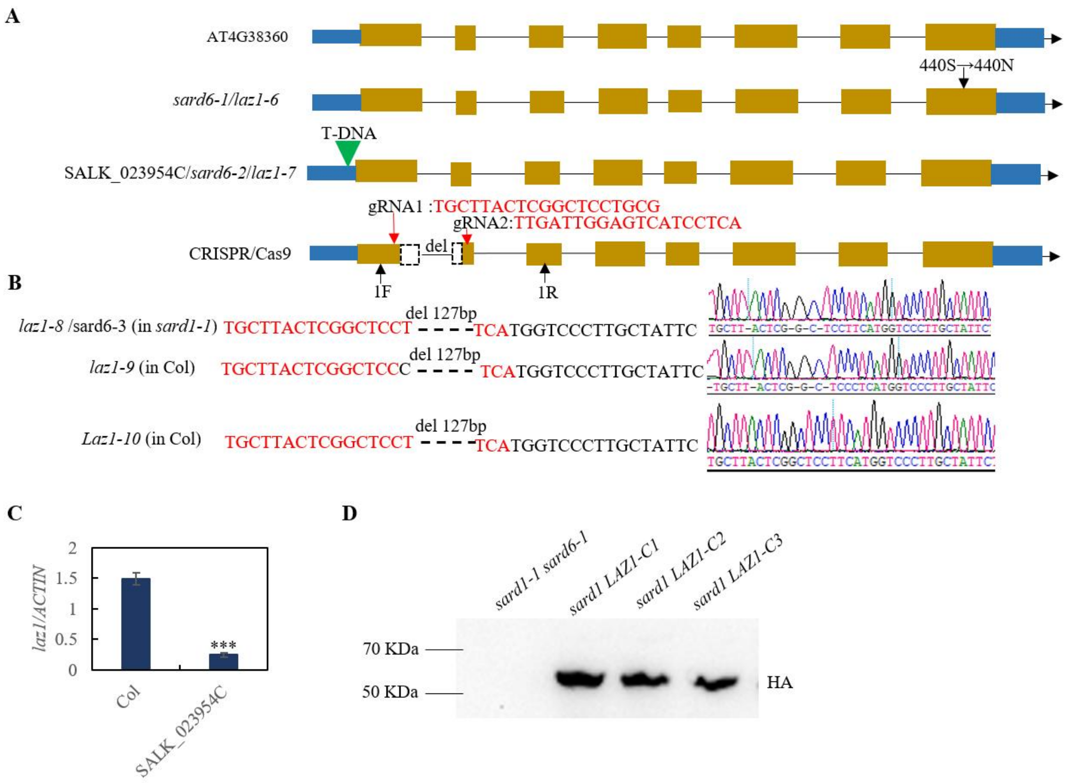

Figure S2. **Knockout and complement analysis of *AT4G38360*.**

A, Schematic diagram of different mutant lines of *AT4G38360*. **B**, Deletion mutation lines of *AT4G38360* generated using CRISPR/Cas9 system. Red bases are the gRNA sequences, the dotted lines indicate the missing bases. **C**, Expression of *LAZ1* in Col and SALK_023954C (*sard6-2*/*laz1-7*). Data were normalized relative to the expression of the *AtActin* gene. Error bars means ± SD of 3 biological replicates. **D**, Detection of protein levels of LAZ1 in complementation lines of *LAZ1* in *sard1-1 sard6-1* using Western blot.

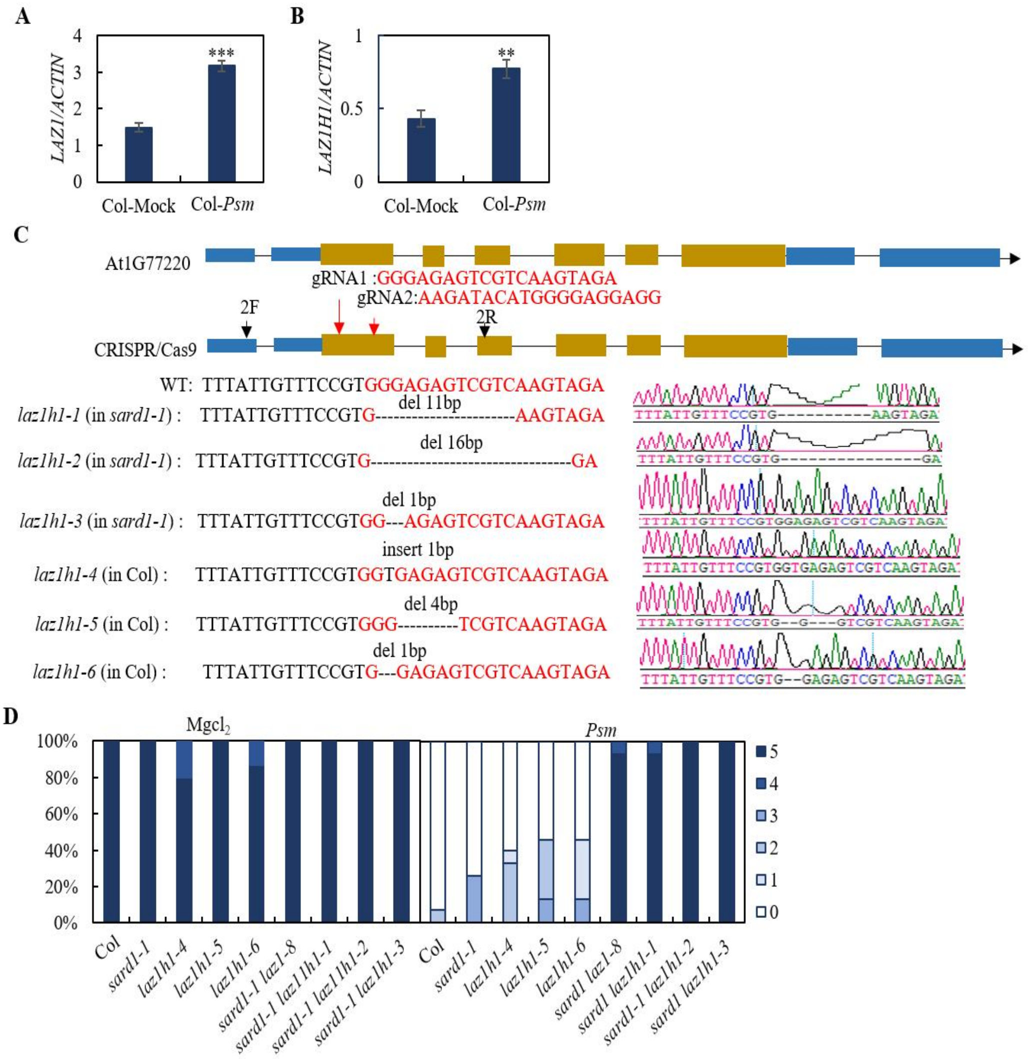

Figure S3. **SAR validation for *laz1h1* mutants in Col and *sard1-1* backgrounds generated using CRISPR/Cas9 system**

A-B, Expression of *LAZ1* and *LAZ1H1*. Total RNA was extracted from the leaves of 3-week-old plants 2d after infiltration with *Psm* ES4326 (OD_{600} = 0.001) or 10 mM $MgCl_2$ (mock). Data were normalized relative to the expression of the *AtActin* gene. Error bars means ± SD of 3 biological replicates. Statistical significance was determined by Student's *t* test. **C**, Schematic diagram of mutation lines of *AT1G77220* generated using CRISPR/Cas9 system. Red bases are the gRNA sequences, the dotted lines indicate the missing bases. **D**, SAR phenotypic statistics of wild-type Col, *sard1-1*, *laz1h1-4*, *laz1h1-5*, *laz1h1-6*, *sard1-1 laz1-8*, *sard1-1 laz1h1-1*, *sard1-1 laz1h1-2* and *sard1-1 laz1h1-3* plants.

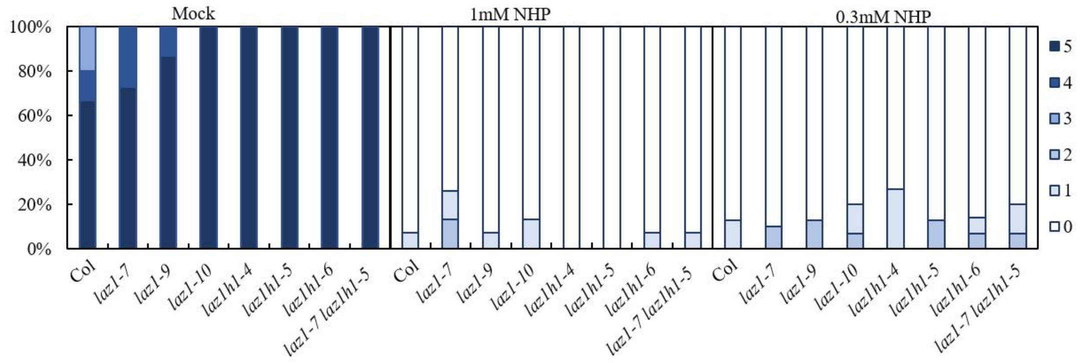

FigureS4. **NHP-induced immunity in *Laz1* and *Laz1H1* mutants**

Phenotypic statistics of NHP-induced immunity against *Hpa* Noco2 in wild-type Col, *laz1-7*, *laz1-9*, *laz1-10*, *laz1h1-4*, *laz1h1-5*, *laz1h1-6* single mutant and *laz1-7 laz1h1-5* double mutant plants. Two primary leaves of 3-week-old plants were infiltrated with 1mM NHP, 0.3mM or ddH$_2$O (mock) two days before the plants were sprayed with *Hpa* Noco2 spore suspension (50,000/mL in water). Disease symptoms were evaluated 7 days post-inoculation using the disease rating scores described in Figure 1B.

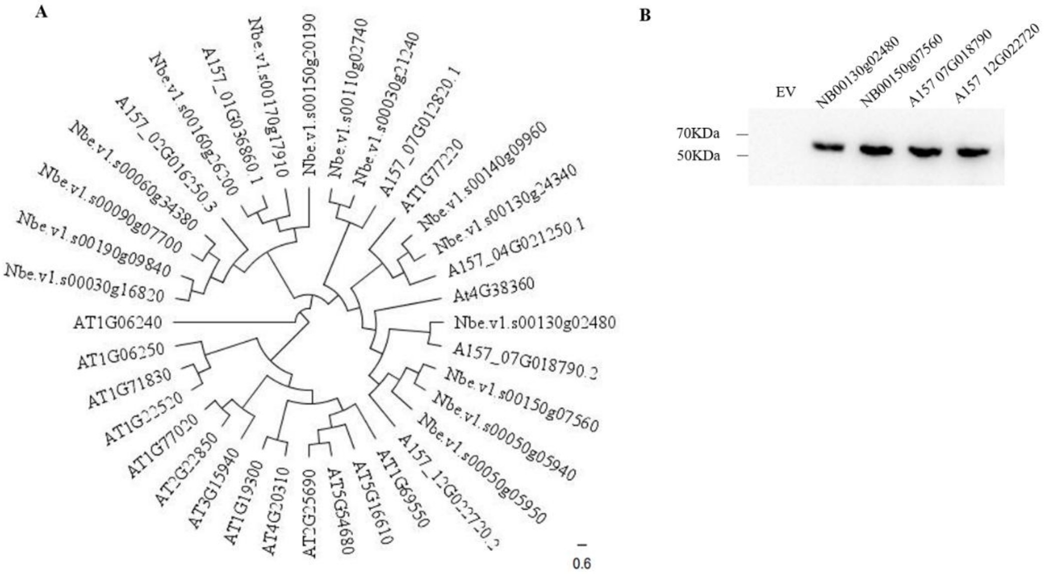

Figure S5. Phylogenetic analysis the homologous of *LAZ1*.

A, Evolutionary analysis of *LAZ1* homologous genes in *Arabidopsis*, *N. benthamiana* and potato. **B**, Western Blot analysis the proteins of *NB00130g02480*, *NB00150g07560*, *A157_12G022720* and *A157_07G018790* transient expression in *Nb* leaves.
